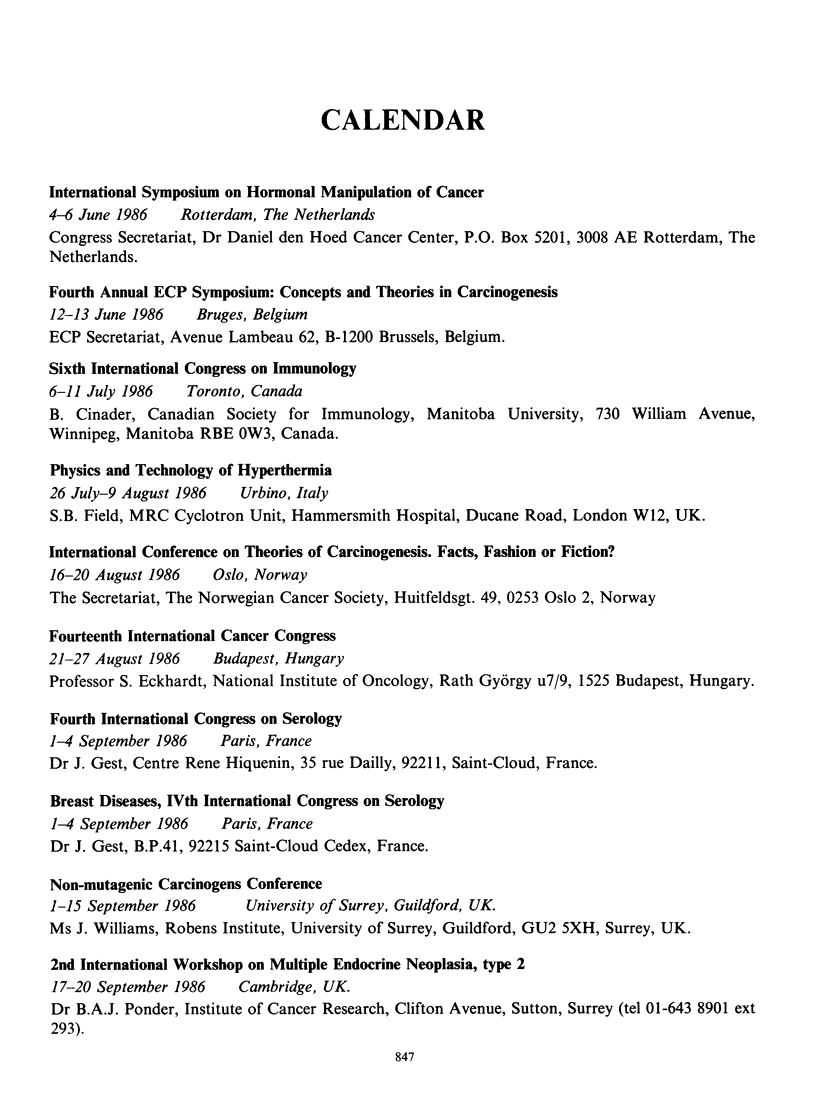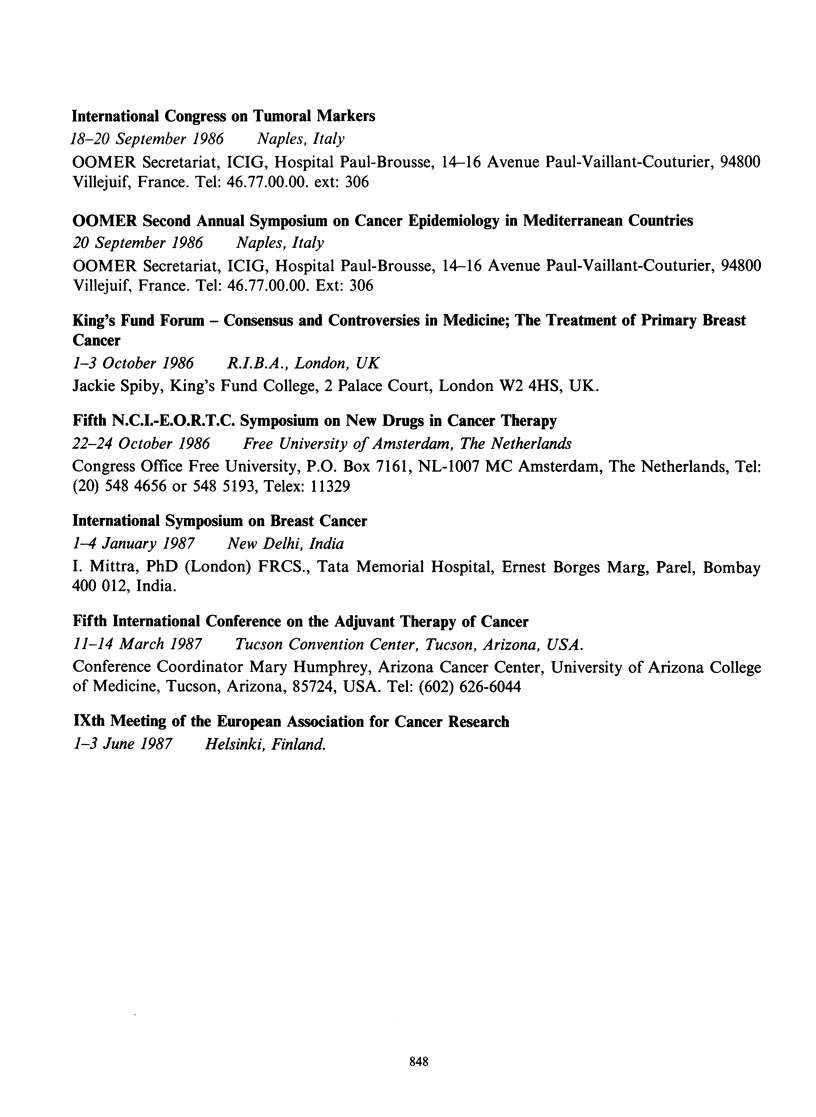# Calendar

**Published:** 1986-06

**Authors:** 


### CALENDAR

**International Symposium on Hormonal Manipulation of Cancer**
*4–6 June 1986  Rotterdam, The Netherlands*
Congress Secretariat, Dr Daniel den Hoed Cancer Center, P.O. Box 5201, 3008 AE Rotterdam, The Netherlands.

**Fourth Annual ECP Symposium: Concepts and Theories in Carcinogenesis**
*12–13 June 1986  Bruges, Belgium*
ECP Secretariat, Avenue Lambeau 62, B-1200 Brussels, Belgium.

**Sixth International Congress on Immunology**
*6–11 July 1986  Toronto, Canada*
B. Cinader, Canadian Society for Immunology, Manitoba University, 730 William Avenue, Winnipeg, Manitoba RBE 0W3, Canada.

**Physics and Technology of Hyperthermia**
*26 July–9 August 1986  Urbino, Italy*
S.B. Field, MRC Cyclotron Unit, Hammersmith Hospital, Ducane Road, London W12, UK.

**International Conference on Theories of Carcinogenesis. Facts, Fashion or Fiction?**
*16–20 August 1986  Oslo, Norway*
The Secretariat, The Norwegian Cancer Society, Huitfeldsgt. 49, 0253 Oslo 2, Norway

**Fourteenth International Cancer Congress**
*21–27 August 1986  Budapest, Hungary*
Professor S. Eckhardt, National Institute of Oncology, Rath György u7/9, 1525 Budapest, Hungary.

**Fourth International Congress on Serology**
*1–4 September 1986  Paris, France*
Dr J. Gest, Centre Rene Hiquenin, 35 rue Dailly, 92211, Saint-Cloud, France.

**Breast Diseases, IVth International Congress on Serology**
*1–4 September 1986  Paris, France*
Dr J. Gest, B.P.41, 92215 Saint-Cloud Cedex, France.

**Non-mutagenic Carcinogens Conference**
*1–15 September 1986  University of Surrey, Guildford, UK.*
Ms J. Williams, Robens Institute, University of Surrey, Guildford, GU2 5XH, Surrey, UK.

**2nd International Workshop on Multiple Endocrine Neoplasia, type 2**
*17–20 September 1986  Cambridge, UK.*
Dr B.A.J. Ponder, Institute of Cancer Research, Clifton Avenue, Sutton, Surrey (tel 01-643 8901 ext 293).

**International Congress on Tumoral Markers**

*18–20 September 1986 Naples, Italy*

OOMER Secretariat, ICIG, Hospital Paul-Brousse, 14–16 Avenue Paul-Vaillant-Couturier, 94800 Villejuif, France. Tel: 46.77.00.00. ext: 306

**OOMER Second Annual Symposium on Cancer Epidemiology in Mediterranean Countries**

*20 September 1986 Naples, Italy*

OOMER Secretariat, ICIG, Hospital Paul-Brousse, 14–16 Avenue Paul-Vaillant-Couturier, 94800 Villejuif, France. Tel: 46.77.00.00. Ext: 306

**King's Fund Forum – Consensus and Controversies in Medicine; The Treatment of Primary Breast Cancer**

*1–3 October 1986 R.I.B.A., London, UK*

Jackie Spiby, King's Fund College, 2 Palace Court, London W2 4HS, UK.

**Fifth N.C.I.-E.O.R.T.C. Symposium on New Drugs in Cancer Therapy**

*22–24 October 1986 Free University of Amsterdam, The Netherlands*

Congress Office Free University, P.O. Box 7161, NL-1007 MC Amsterdam, The Netherlands, Tel: (20) 548 4656 or 548 5193, Telex: 11329

**International Symposium on Breast Cancer**

*1–4 January 1987 New Delhi, India*

I. Mittra, PhD (London) FRCS., Tata Memorial Hospital, Ernest Borges Marg, Parel, Bombay 400 012, India.

**Fifth International Conference on the Adjuvant Therapy of Cancer**

*11–14 March 1987 Tucson Convention Center, Tucson, Arizona, USA.*

Conference Coordinator Mary Humphrey, Arizona Cancer Center, University of Arizona College of Medicine, Tucson, Arizona, 85724, USA. Tel: (602) 626-6044

**IXth Meeting of the European Association for Cancer Research**

*1–3 June 1987 Helsinki, Finland.*